# Halting HIV/AIDS with avatars and havatars: a virtual world approach to modelling epidemics

**DOI:** 10.1186/1471-2458-9-S1-S13

**Published:** 2009-11-18

**Authors:** Richard Gordon, Natalie K Björklund, Robert J Smith?, Eluemuno R Blyden

**Affiliations:** 1 Department of Radiology, University of Manitoba, GA216, HSC, 820 Sherbrook Street, Winnipeg, MB R3A 1R9, Canada; 2 Silver Bog Research Inc., 350 Inkster Blvd., Winnipeg MB R2W 0K3, Canada; 3 Department of Mathematics & Faculty of Medicine, The University of Ottawa, 585 King Edward Drive, Ottawa, ON K1N 6N5, Canada; 4 AfriVax, Inc., 613 19th Ave. E. Suite 101, Seattle WA 98112, USA

## Abstract

**Background:**

A major deficit of all approaches to epidemic modelling to date has been the need to approximate or guess at human behaviour in disease-transmission-related contexts. Avatars are generally human-like figures in virtual computer worlds controlled by human individuals.

**Methods:**

We introduce the concept of a "havatar", which is a (human, avatar) pairing. Evidence is mounting that this pairing behaves in virtual contexts much like the human in the pairing might behave in analogous real-world contexts.

**Results:**

We propose that studies of havatars, in a virtual world, may give a realistic approximation of human behaviour in real-world contexts. If the virtual world approximates the real world in relevant details (geography, transportation, etc.), virtual epidemics in that world could accurately simulate real-world epidemics. Havatar modelling of epidemics therefore offers a complementary tool for tackling how best to halt epidemics, including perhaps HIV/AIDS, since sexual behaviour is a significant component of some virtual worlds, such as Second Life.

**Conclusion:**

Havatars place the control parameters of an epidemic in the hands of each individual. By providing tools that everyone can understand and use, we could democratise epidemiology.

## Background

Epidemiology has always had control of epidemics as its goal. Ever since we discovered that mosquitoes transmit malaria, we have sought points of control, from eradication of mosquito larvae, to screening of windows and beds, to the development of vaccines, to more abstract points of control, such as keeping the reproductive rate of an epidemic, R_0_, below 1 [1]. With HIV/AIDS, like the cartoon character Pogo, "We have met the enemy and he is us" [2]. While for decades we have hoped that an effective vaccine is just around the corner [3], "HIV-vaccine research [is] a field that has endured a Sisyphean onslaught of disappointments" [4], so that, for now, it is our own behaviour that brings or avoids HIV/AIDS. We have to look within, rather than to externalities, for control points. Virtual worlds potentially offer unprecedented levels of access to the internal dynamics of behaviour, complete with a new set of ethical issues.

Epidemiology modelling has generally followed two major approaches: the continuous and the discrete. In the continuous (deterministic) approach, we use differential or partial differential equations, characterised by lumped or aggregated variables and presumed constants relating them. We try to predict the variables, such as the number of susceptible or infected people, given constants, such as the rate of transmission [5,6]. In the discrete (individual) approach [7], we divide the population into individuals, and try to model their interactions, sometimes describing them as occurring on a social network, which is a discrete structure in the sense of graph theory. This discrete structure can take the extreme of complete representation of urban geography and transportation [8]. An analogy may be made with the physics of fluids. We may try to solve the continuous equations of fluid flow, as in computational fluid dynamics [9], or we may try to simulate the behaviour of all the individual molecules in the fluid and their interactions with those they come in contact with, as in Monte Carlo molecular dynamics [10-14].

Stochastic process modelling [15] represents a compromise, in which we attempt to use aggregated parameters when approximating large populations, but add in "noise" to represent individual variations. Stochastic models generally agree with deterministic models when the disease is endemic, but may differ significantly when the disease is close to extinction or just starting. Stochastic effects tend to drive outcomes that are near zero to actual extinction, which would theoretically take an infinite amount of time in deterministic models.

Certain control points, i.e., parameters that we think we can manipulate to alter the course of an epidemic, are evident with HIV/AIDS. For example, it has long been known that HIV/AIDS could be significantly controlled with condoms, if only we could get enough people to use them [16-21]. Thailand took this approach, which met with great success [22,23], but continual attention is needed as behaviour changes [24]. For example, the growth of intravenous drug use provides a route of transmission for which condoms are ineffective, exasperated by the observation that condom use is difficult to promote amongst intravenous drug users for protecting their sexual partners [25]. For HIV/AIDS, we must therefore have multiple control points.

Two observations, however, make the multiple control point approach dubious [26]. First is the observation of, at best, a weak correlation between the aggregated parameters of the continuous approach, that cannot be dismissed as mere statistical inaccuracy in estimating parameters [27]. In other words, when we guess at what factors are important (such as aggregating people into certain categories) and how they should affect one another (such as presuming which aggregated groups mate with which other groups), the predictive value of our models proves to be quite poor, or downright wrong. More refined continuous models help to some extent in unravelling deeper causal relations and improving correlations, but the very concept that all people in one group randomly mate with people in another group is obviously a poor approximation to how, where, when and how often they actually pair up. Poisson statistics are implicitly assumed, which are themselves but a crude approximation to the time course of human behaviour.

The second doubt [26] about the multiple-control-point approach is the dynamic nature of networks of human sexual behaviour when controls are applied. For example, police action may close down one venue for sex work, but an alternative arises to replace it. Thus, shifting behaviour and "hidden groups" [28,29] not foreseen by field workers make control points a matter of guesswork. One hope is that a higher understanding of dynamic networks as complex systems may give us new points of control [26], but how to observe changing networks of behaviour with limited field work resources is problematic.

In a way, these efforts to refine different models make them approach one another. We discretise the continuous approach, by subdividing aggregated parameters, or smooth the discrete approach, by looking at the overall behaviour of networks, their so-called emergent features. But do they thereby better approximate the real world?

While we try to empower people through education, stigma reduction, harm reduction, free condoms, needle exchange programs, etc., the role of the individual in halting the HIV/AIDS epidemic is basically passive with these approaches. We somehow hope that when they choose a mate, engage a sex worker, etc., individuals will modify their behaviour in a way that reduces or eliminates their contribution to the dynamics of the epidemic. When they fail to do as we hope, we attribute this to inadequate education or empowerment, or the heat of the moment. We expect that, somehow, our grand view of the epidemic, as professional epidemiologists or modellers, will influence individuals' behaviours and thus affect parameters such as the rate of transmission. Analogous to the continuum between the discrete and the continuous in our models, however, the socio-cultural "view" of the epidemic ranges from the local one of the individual in the epidemic, to that of the global view of scientists, mathematicians, health economists and epidemiologists who actually deal with a lumped or aggregated conceptualization of the epidemic. Even when they are successfully communicated, faith in different views of the epidemic is rarely shared between all concerned. People have different priorities and relate differently to the need for behavioural change - ultimately underpinned by differences in valuations of life and death itself. The consequences are not trivial. The success of science in understanding HIV/AIDS has relied heavily on the willing co-operation of individuals whose view of the epidemic cannot prevent them from interacting with it, such as sex workers and intravenous drug users.

## A new approach to epidemic modelling using havatars

All epidemic modelling to date approximates and second-guesses human behaviour. We would like to propose an alternative way to model HIV/AIDS, that lets each individual person decide on the control parameters for their life, and which makes their actual behaviour part of the modelling. Our suggestion is an old approach, what used to be called "enlightened self-interest" [30]. It assumes, contrary to the "tragedy of the commons" [31], that what is best for the individual from their own point of view will be best for society as a whole. That is, of course, unproven for HIV/AIDS, so what we are proposing here is a new avenue of epidemiological research.

We start with the observation that, in certain virtual worlds, which appear to be those where the participants construct the rules of engagement with one another themselves, there seems to be a correlation between the personality of a person's avatar [32] and their own self [33-35]. For compactness of language, we will define a person:avatar pair as a "havatar", where the "h" stands for "human"; thus, havatar = (human, avatar). The nature of these pairings (i.e., the identity formed) depends on the way the virtual world is set up. Second Life generally has havatars [33], whereas games in which a person has multiple avatars under their control may diminish the correlation or create more complex correlations. The self-constructed or self-selected nature of the rules, landscape, furniture, vehicles, clothing and architecture in Second Life may also invite imitation of each person's real life, making it much more "true to life".

In adult virtual worlds, as in the internet itself, a significant fraction of the interactions between havatars is sexual [36]. HIV/AIDS cannot, of course, be transmitted between avatars, so that sex in a virtual world could be the ultimate in safe sex. Nevertheless, computer viruses have actually been transmitted between avatars, both accidentally and intentionally [37]. One remarkable observation about these social experiments is that havatars undertake behaviours of evasion of the avatar epidemic that are similar to those humans might take in an analogous real life situation. In other words, the equation of the avatar with "one's self" may be quite strong in havatars. One could add visible symptoms, pre- and post-phylactic interventions, simulated funerals, etc., to make quite a visual impact and provide different "views" of the epidemic. Mythologies developed about how to avoid an avatar epidemic [37], which remind one of mythologies about HIV/AIDS, from total denialism [38] to counterproductive practices that infect others [39-41].

Beyond this, each individual has the opportunity of seeing the epidemic at any scale, through statistics gathered by the computer program running the virtual world, creation of maps, communication with and knowledge about other havatars, and by being allowed to run computer simulations of the epidemic themselves. For example, in Second Life, one can examine the profile of any havatar, which includes the names of the groups they belong to, and decide whether or not to become "friends" with them. Such textual, quantitative and visual tools can provide people with objective criteria that relate their own behaviour to an epidemic, and give them opportunities to change that behaviour to avoid being part of it.

Havatars thus present the possibility of a bottom-up approach to epidemic control. They are already being used to plan for emergencies caused by epidemics [42,43], so why not to study actual epidemics? If people identify with their avatars, we could imagine the possibility of everyone taking control of HIV/AIDS in their own lives, based on information about the epidemic attained through a virtual world. This assumes, of course, a strong correlation between real life, person-to-person sex, and the corresponding havatar-to-havatar sex. The latter allows more "positions" than most people are capable of actually doing, and one could assume that people might take more risks as havatars than they would in real life. We know from real life that such additional risk-taking extends the range of experimental opportunity for scientific studies of HIV/AIDS. If epidemiologist havatars could experiment with scenarios themselves, new discoveries about the subtleties of human-virus interaction might be uncovered. Clearly, we need some research on whether the correlations between real and havatar personalities [33] extend to sexual behaviour.

## Discussion

Havatars could be used by epidemiological modellers in a variety of ways. The simplest would be to unleash simulated viruses that can infect avatars, are invisible, have no effects, but whose transmission from one havatar to another can be monitored. This has a large advantage over real-life networks in that the latter require tremendous amounts of fieldwork to uncover. Furthermore, such fieldwork might itself alter the social network. Details of where, when, what acts, and with whom have the general unreliability of self-reporting; fieldwork at best gives a snapshot of a dynamic system, while in a virtual world all this data could be made available on a continuous basis. Waivers that allow tracking of all transactions were signed by all 13 million Second Life participants [44] when they joined [45]. The social networks of havatars observed in a virtual world may prove a better representation of real-world disease transmission networks than any attempt at direct observation of the real world could ever produce.

The research on havatars that we propose raises questions of privacy, ethics and informed consent that are unresolved as of now, but it has been suggested that the general difficulty of a person knowing which other real life person corresponds to a given havatar may keep these problems at bay [46].

The next level of havatar epidemiology research would be to create consequences of disease transmission. In virtual worlds, people react to parameters at many levels, and have the opportunity to grasp a situation as a whole [47]. This is what makes multilevel control by havatars a potentially powerful means of epidemic control, because the person involved has the opportunity to grasp the epidemic in many ways. We can see this operating in the simulated "Whypox" epidemics [37]. Two simple consequences occurred for avatars: they acquired spots on their skins that the person could not remove, and they "sneezed", interjecting the word "achoo" at random in typed conversations between havatars, sometimes requiring retyping of a command that would have initiated an animated sequence. The people behind the avatars reacted strongly to this imposed situation, and changed their behaviour to alleviate or avoid it.

For an HIV/AIDS simulation, we could envision more drastic consequences for havatars, with permission of their human components. For example, most people design their avatars to look good. Simulated HIV/AIDS might make them look ghastly, with weight loss, etc., cause them to interrupt their behaviour to take antiretroviral drugs, or even get too sick to do much and die. This might lead to stigma, formation of ALWA groups (Avatars Living With AIDS), etc. Analogous avatar effects have been simulated for neurological conditions [48]. Economic losses [49,50], such as (simulated) drug costs, could also be of consequence not only to the avatar and its accoutrements, but also to the real-life person who has to pay for some of them with real money [51]. Thus, with such an epidemiological experiment, we would start crossing the virtual/real-world boundary. This boundary crossing can clearly be seen in the behaviour of real life married couples who enter a virtual world [52].

The third level of epidemiological research would fully cross the virtual world/real-world boundary. This is already being done with computer-controlled sex toys that can be controlled by other havatars [53], and could be regarded as an extension of the search for self-knowledge, which often involves sexual behaviour [54]. Of course, so long as these toys aren't shared between real people, real HIV won't be transmitted. But if the avatars can be computer infected, their people might react accordingly. This raises the general question of whether internet sex, like phone sex, will become sufficiently widespread, perhaps because of these remotely controllable haptic devices, to impact and cut into real-world sex work (Joel Kettner, personal communication). The argument that condom availability promotes promiscuous behaviour rapidly lost out to the perception that condoms prevent disease transmission, and, at least in the case of Thailand, promotion of condom use and HIV/AIDS awareness led to a decline in sex work [24]. Similarly, the current notion that the Internet facilitates sexual contacts may lose out to idea that the Internet prevents real disease transmission.

Whether havatar sex will have an impact on HIV transmission between real humans is of course not yet known. When one havatar encounters another, they can examine each other's "profiles", which include, for example, listings of the groups they belong to. The record could show simulated HIV status as one of the consequences in the virtual world. Attempts to do anything similar in the real world are limited so far. Sex clubs and pornography actors have tried to restrict membership to HIV-free people, and a few cases of failure to divulge one's HIV status have hit the courts, but how this plays out in daily life is uncertain, and obviously of insufficient impact to halt the HIV/AIDS epidemic. Transferring codes of ethics developed in virtual worlds to real life is problematic, though even in the freewheeling atmosphere of Second Life, the reverse has already occurred in terms of gambling and banking [55]. However, havatars give us an interesting tool with which to explore these options for epidemic control.

It will be argued against this approach that the cost of Internet access is beyond the poor populations of the world where HIV/AIDS flourishes most, and that the havatar population is thus a biased sampling of the human population. However, if we consider the rate of adoption of cell phones [56] or programs to introduce rugged, hand- and solar-powered, internet-connectable computers at low cost worldwide [57], the democratising influence of virtual worlds [58], and the widespread use of the internet to make sexual contacts [59], this leapfrogging might occur quite rapidly.

## Conclusion

This paper is part of an effort to develop a worldwide model of the HIV/AIDS epidemic and how to control and stop it, beginning with the MITACS http://www.mitacs.math.ca/ OptAIDS Workshop July 29, 2008 in Toronto. The goal, WHAM, the World Halting AIDS Model, [60,61] might be just a grand epidemiology simulation in the deterministic and/or stochastic moulds. However, if we let the people of the world simulate themselves through havatars, we may collectively see our way past this unfortunate pandemic, and who knows what else that may lie ahead. We may need a new approach, since, for all the work that has been done on trying to control the HIV/AIDS epidemic, it is our impression that, for the most part, we have just helplessly watched it grow.

The aim of epidemiological modelling has always been to gain control of epidemics. HIV/AIDS is an epidemic that is very much depends on the decisions made by individuals. Controlling them, telling people what to do for their own or the greater, common good, has proven to be a difficult and sometimes thankless task. Perhaps what we need to do is examine the relationship between the modeller and those modelled. Havatars give us an opportunity to reverse that relationship and place the control parameters in the hands of each individual. The modeller can enable this democratisation of epidemiology by providing tools for global and other perspectives that everyone can understand and use.

## Competing interests

The authors declare that they have no competing interests.

## Authors' contributions

RG wrote the first draft. All authors read and approved the final manuscript.
